# Supplementary figures and images for: Gene expression of benthic amphipods (genus: Diporeia) in relation to a circular ssDNA virus across two Laurentian Great Lakes
Source: PeerJ. 2017 Sep 26;5:e3810. doi: 10.7717/peerj.3810 (PMC5621510; doi:10.7717/peerj.3810)

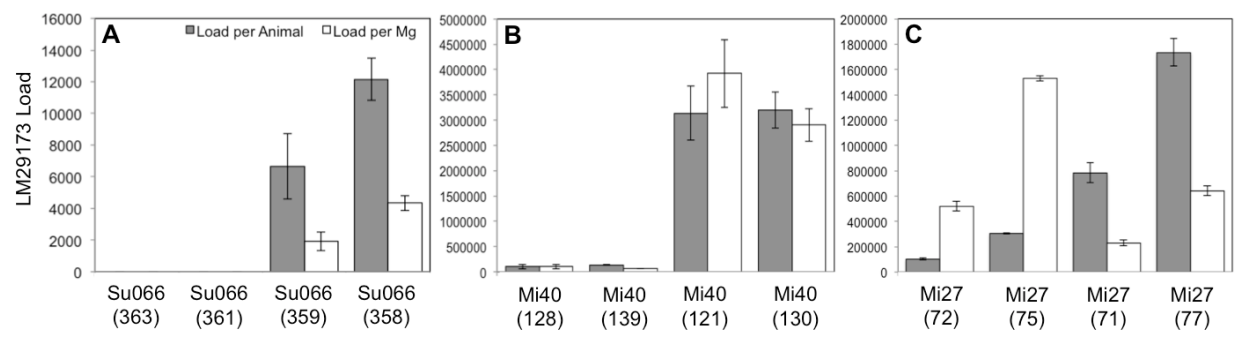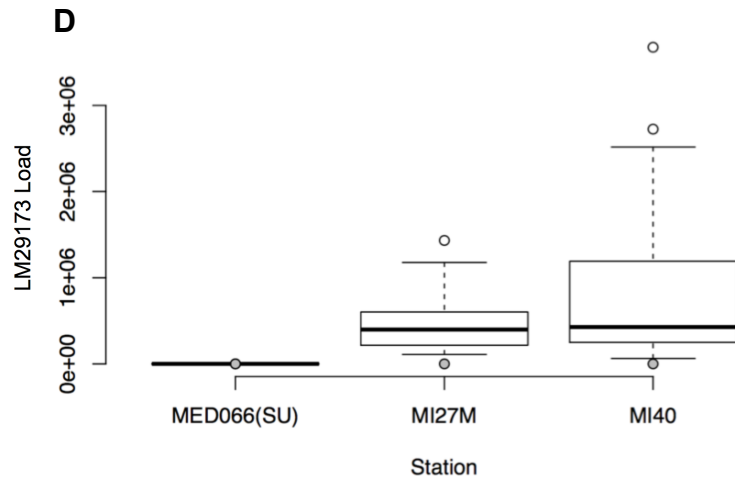

Supplement: Supplemental Information 1 — (A–C) Load of LM29173 copy number animal−1 or mg−1 (± 1SE between quadruple technical replicates) in organisms from stations Superior 066 (A, Su066), Michigan 40 (B, Mi40), and Michigan 27 (C, Mi27). (D) Boxplot illustrating distribution of viral load among amphipods from three stations. Outliers (< average and > average LM29173 genome copies animal−1) were selected for transcriptome preparation. [file peerj-05-3810-s001.pdf]

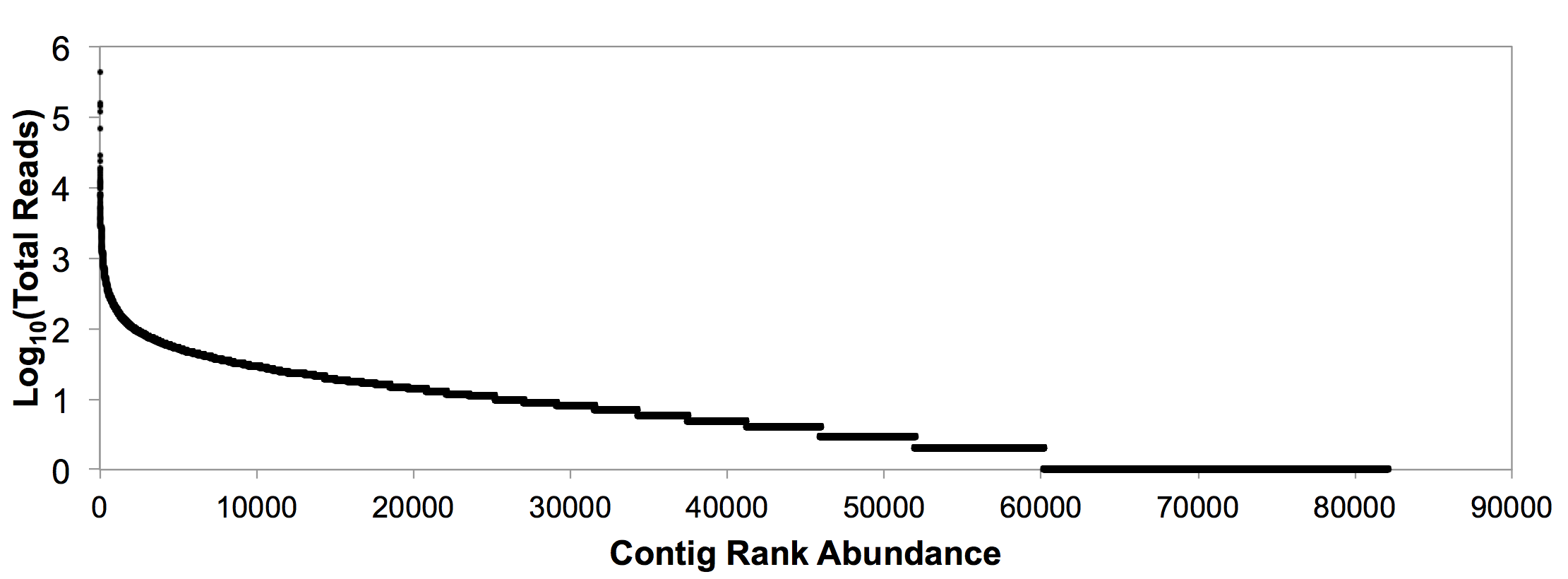

Supplement: Supplemental Information 2 [file peerj-05-3810-s002.png]

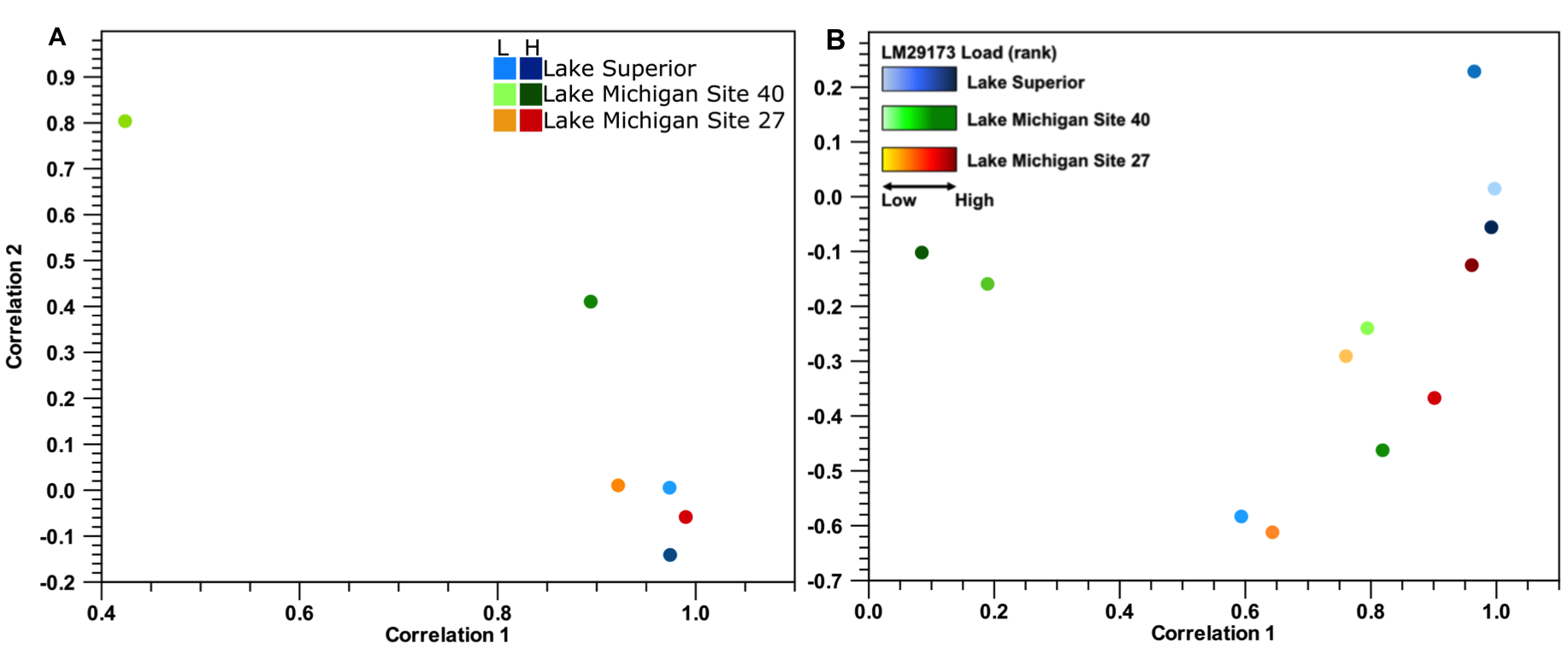

Supplement: Supplemental Information 3 — Plots were generated via CLC workbench (v. 8.5.1, Qiagen, Hilden, Germany) with default parameters. [file peerj-05-3810-s003.png]

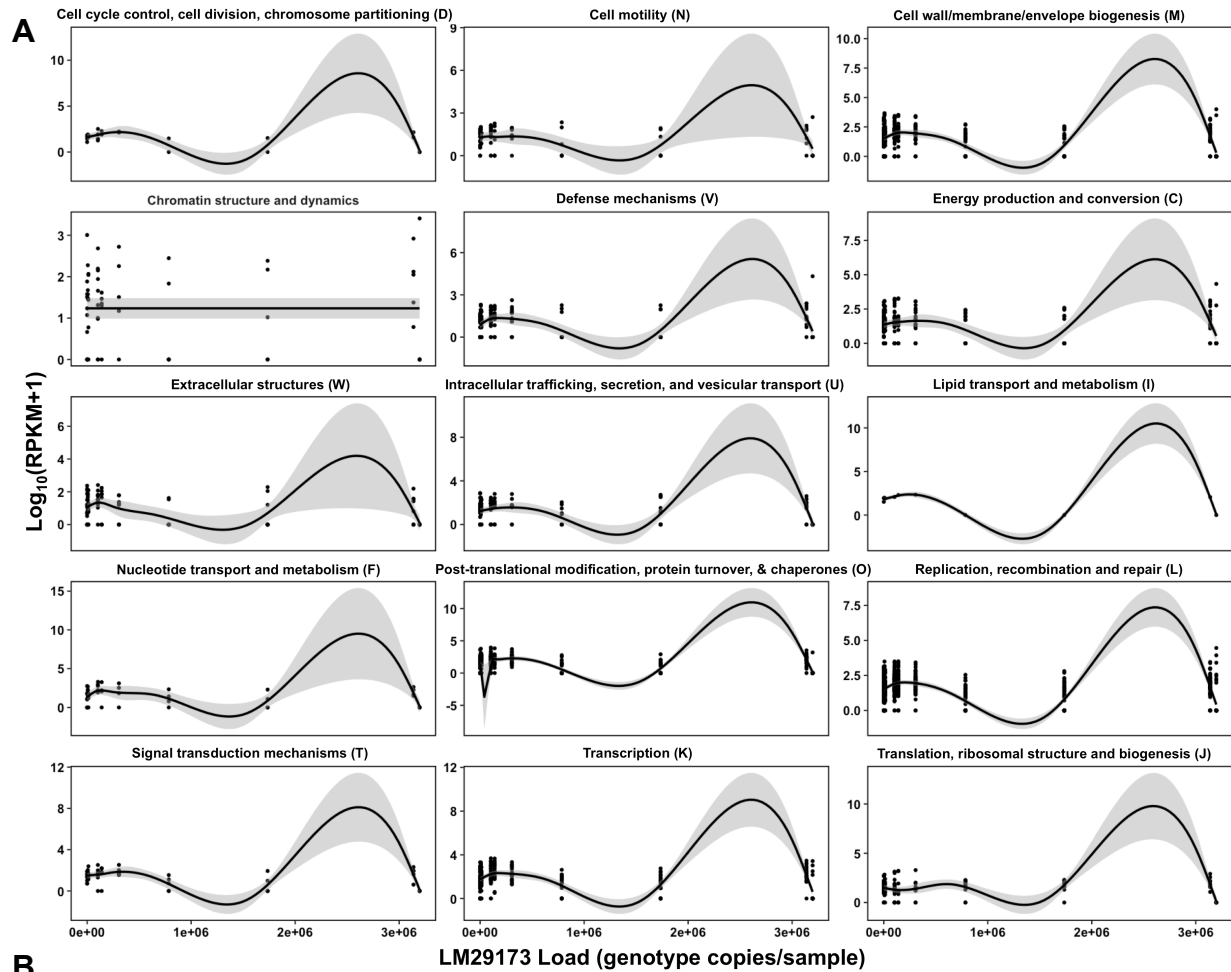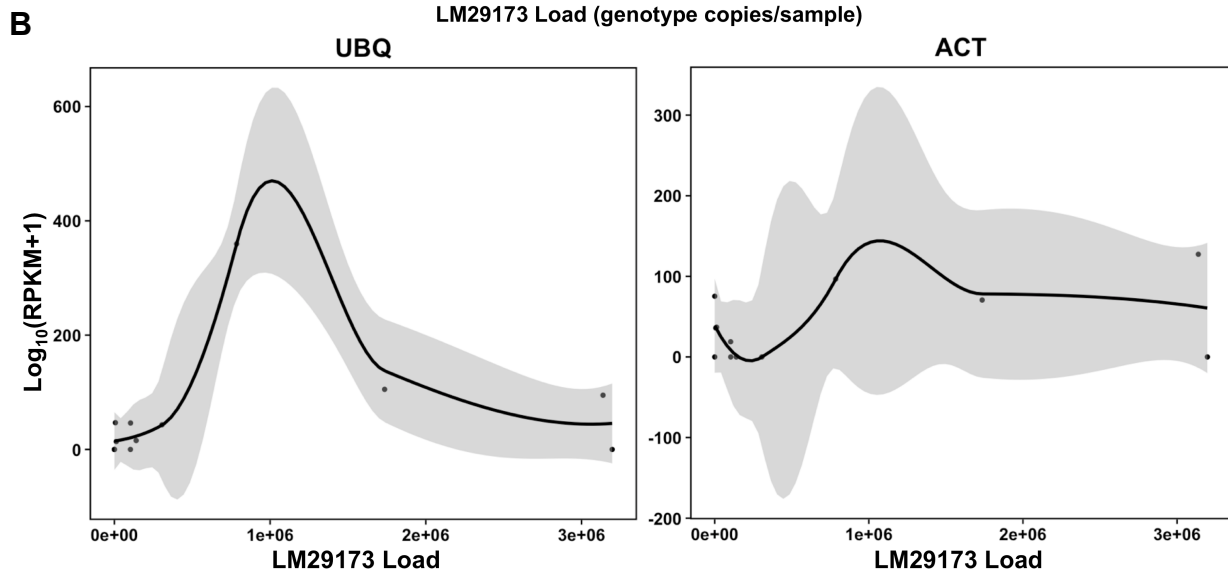

Supplement: Supplemental Information 4 — (A) Density estimation distributions depicting expression (Log10(RPKM + 1)) of contigs associated with 15 KOG classes at a range of viral loads (LM29173 load organism−1; n = 12 transcriptomes). (B) Density estimation distributions of RT-qPCR target contigs UBQ and ACT depicting expression (Log10(RPKM + 1)) at a range of viral loads (LM29173 load organism−1; n = 12 transcriptomes). [file peerj-05-3810-s004.pdf]

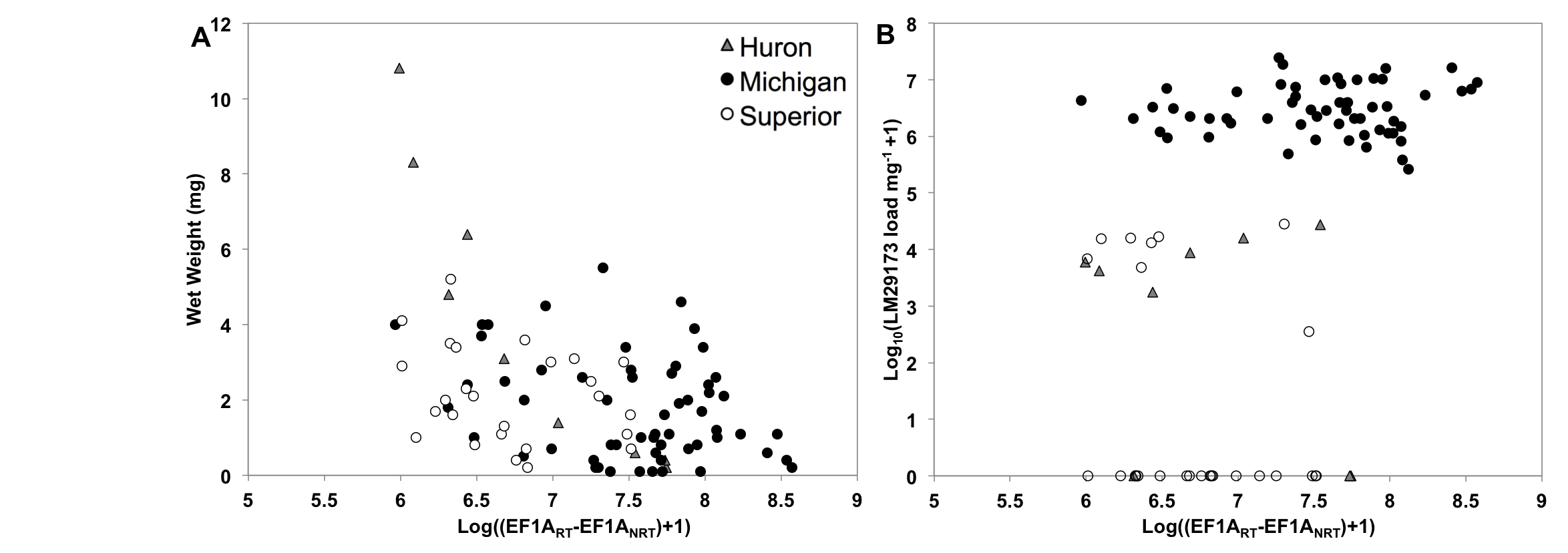

Supplement: Supplemental Information 5 — EF1A expression is reported as the quantitated difference between reverse transcribed and non-reverse transcribed RNA extractions. i.e. EF1ART–EF1ANRT, where RT and NRT indicate samples that have been reverse transcribed via Superscript III (Invitrogen, Carlsbad, CA, USA) or not reverse transcribed (no-RT control), respectively. [file peerj-05-3810-s005.png]
