# Supplementary material for: Gene expression of benthic amphipods (genus: Diporeia) in relation to a circular ssDNA virus across two Laurentian Great Lakes
Source: PeerJ. 2017 Sep 26;5:e3810. doi: 10.7717/peerj.3810 (PMC5621510; doi:10.7717/peerj.3810)
Supplement: Supplemental Information 6 — Contigs are grouped by putative function: (A) immune response regulation, (B) antimicrobial peptides, (C) phagocytosis, (D) protease/chitinase, (E) JAK/STAT pathway, JNK pathway, (F) toll pathway, (G) prophenoloxidase system, (H) stress/oxidative damage response, (I) transcription factors, (J) signaling, post-translational modification, (K) cell structure, mobility, and intracellular trafficking, (L) receptors, and (M) cell cycle. Image generated by web-based visualization tool, Morpheous (Broad Institute, Cambridge, MA, USA). [file peerj-05-3810-s006.pdf]

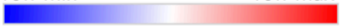

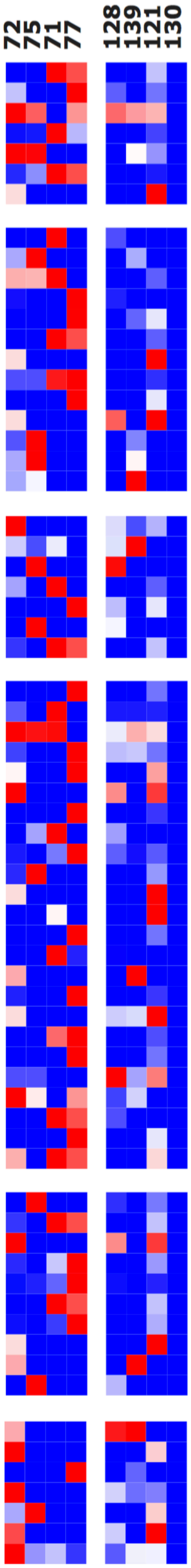

	Contig ID	Accession	Putative Function	E-value	Host
A	DN7143c1g1i1	XP392454.2	alpha-1-inhibitor 3	8.000e-19	*Apis mellifera*
	DN37192c0g1i1	XP392454.2	alpha-1-inhibitor 3	4.000e-9	*Apis mellifera*
	DN10758c0g1i3	AIZ03630.1	cactin	3.000e-7	*Litopenaeus vannamei*
	DN19783c0g1i1	AJD07838.1	calcineurin subunit A	2.000e-7	*Eriocheir sinensis*
	DN8923c0g1i1	AGS12619.1	NF-kB	0.000003	*Tigriopus japonicus*
	DN10894c0g1i1	AGA16579.1	QM protein	2.000e-111	*Litopenaeus vannamei*
	DN20358c0g1i1	AFU60735.1	Relish 1b	0.000009	*Fenneropenaeus chinensis*
B	DN25682c0g1i1	AHB63227.1	argonaute 2	6.000e-18	*Penaeus monodon*
	DN9649c0g2i2	AAO39550.1	argonaute 2	0.000009	*Drosophila melanogaster*
	DN22598c0g1i1	ACU25384.1	crustin 3	6.000e-15	*Panulirus japonicus*
	DN23433c0g1i1	XP001606025.1	MIB like protein	5.000e-7	*Nasonia vitripennis*
	DN22473c1g1i1	CCE46010.1	putative DEAD-box ATP-dependent RNA helicase*	1.000e-8	*Cancer pagurus*
	DN37858c0g1i1	CCE46010.1	putative DEAD-box ATP-dependent RNA helicase*	2.000e-33	*Cancer pagurus*
	DN5585c0g2i1	CCE46010.1	putative DEAD-box ATP-dependent RNA helicase*	2.000e-8	*Cancer pagurus*
	DN36349c0g1i1	CCE46010.1	putative DEAD-box ATP-dependent RNA helicase*	3.000e-83	*Cancer pagurus*
	DN15339c0g1i1	ACF28464.1	single whey acidic protein domain-containing protein	0.000008	*Penaeus monodon*
	DN36978c0g1i1	BAD99524.1	VASA RNA helicase	0.000003	*Moina macrocopa*
	DN46355c0g1i1	BAD99524.1	VASA RNA helicase	0.000002	*Moina macrocopa*
	DN21315c0g1i1	BAD99524.1	VASA RNA helicase	0.000001	*Moina macrocopa*
	DN2900c0g1i1	BAD99524.1	VASA RNA helicase	1.000e-9	*Moina macrocopa*
C	DN5865c0g2i1	AFK91931.1	185/333	4.000e-7	*Heliocidaris erythrogramma*
	DN4709c0g1i1	ACZ26466.1	Dscam isoform	1.000e-7	*Litopenaeus vannamei*
	DN7879c0g1i1	ACC65888.1	Dscam isoform	0.000008	*Daphnia pulex*
	DN52194c0g2i1	ACC65888.1	Dscam isoform	0.000002	*Daphnia pulex*
	DN22448c0g1i1	ACC65888.1	Dscam isoform	0.000003	*Daphnia pulex*
	DN41253c0g1i1	ACC65888.1	Dscam isoform	0.000005	*Daphnia pulex*
	DN12714c0g1i1	AHH86070.1	Dscam, partial	8.000e-7	*Cherax quadricarinatus*
D	DN16374c0g1i1	AII16489.1	26S protease regulatory subunit 10B, partial	1.000e-7	*Paracyclopina nana*
	DN11772c0g2i2	AII16487.1	26S protease regulatory subunit 4, partial	2.000e-17	*Paracyclopina nana*
	DN6228c0g1i2	JAJ21823.1	brain chitinase and chia	0.000004	*Daphnia magna*
	DN53134c0g1i1	JAJ57219.1	brain chitinase and chia	6.000e-7	*Daphnia magna*
	DN12189c0g2i1	JAJ57471.1	brain chitinase and chia	2.000e-7	*Daphnia magna*
	DN937c0g1i2	JAJ58565.1	brain chitinase and chia	0.000003	*Daphnia magna*
	DN10624c0g1i2	JAJ59664.1	brain chitinase and chia	0.000002	*Daphnia magna*
	DN11002c0g2i2	JAM72468.1	brain chitinase and chia	0.000002	*Daphnia magna*
	DN5768c0g1i1	JAI92625.1	brain chitinase and chia, partial	0.00001	*Daphnia magna*
	DN5742c0g1i1	JAN16453.1	brain chitinase and chia, partial	2.000e-7	*Daphnia magna*
	DN38929c0g1i1	AGT29851.1	cathepsin B	2.000e-25	*Eriocheir sinensis*
	DN10684c0g1i1	AFC60662.1	chitinase	2.000e-7	*Pandalopsis japonica*
	DN43228c0g1i1	ACG60513.1	chitinase 1 precursor	2.000e-38	*Litopenaeus vannamei*
	DN22703c0g1i1	ACG60513.1	chitinase 1 precursor	0.000001	*Litopenaeus vannamei*
	DN28844c0g1i1	ADG22162.1	chitinase 1 precursor, partial	1.000e-2	*Penaeus monodon*
	DN4131c0g1i1	AHL28109.1	chitinase 4	0.000001	*Macrobrachium nipponense*
	DN10677c0g2i1	AKP18003.1	chitinase 4	0.000004	*Eriocheir sinensis*
	DN45465c0g1i1	XP018011674.1	probable chitinase 3 isoform X1*	5.000e-15	*Hyalella azteca*
	DN7991c0g1i1	JAL35312.1	putative Brain chitinase and chia	3.000e-11	*Daphnia magna*
	DN10384c0g4i1	JAN60243.1	putative Brain chitinase and chia	1.000e-8	*Daphnia magna*
	DN9145c0g1i1	JAN60243.1	putative Brain chitinase and chia	3.000e-8	*Daphnia magna*
	DN19356c0g1i1	CDW36302.1	putative chitinase 3like	4.000e-11	*Lepeophtheirus salmonis*
	DN43573c0g1i1	CDW49744.1	putative chitinase 3like	1.000e-15	*Lepeophtheirus salmonis*
	DN138c0g1i1	AGT03506.1	zinc proteinase, partial	2.000e-7	*Procambarus clarkii*
E	DN44455c0g1i1	XP391941.2	agrin - JNK	1.000e-9	*Apis mellifera*
	DN48524c0g1i1	XP001608105.1	CG7586-PA - JAK/STAT	6.000e-2	*Nasonia vitripennis*
	DN50206c0g1i1	XP001608105.1	CG7586-PA - JAK/STAT	2.000e-9	*Nasonia vitripennis*
	DN10723c0g1i1	ACYPI004970	domeless 2 - JAK/STAT	0.000007	*Acyrthosiphon pisum*
	DN11057c0g2i1	ACYPI004970	domeless 2 - JAK/STAT	9.000e-7	*Acyrthosiphon pisum*
	DN9026c0g1i1	ACYPI004970	domeless 2 - JAK/STAT	5.000e-8	*Acyrthosiphon pisum*
	DN6356c0g1i1	ACYPI004970	domeless 2 - JAK/STAT	0.000009	*Acyrthosiphon pisum*
	DN1068c0g2i1	ACYPI004970	domeless 2 - JAK/STAT	0.000009	*Acyrthosiphon pisum*
	DN1935c0g1i2	ACYPI004970	domeless 2 - JAK/STAT	4.000e-7	*Acyrthosiphon pisum*
	DN1015c0g1i1	ACYPI004970	domeless 2 - JAK/STAT	0.000004	*Acyrthosiphon pisum*
F	DN2757c0g2i1	XP 317365.4	CLIPE1	9.000e-7	*Anopheles gambiae*
	DN34210c0g1i1	NP523776.1	POSH	3.000e-9	*Drosophila melanogaster*
	DN44408c0g1i1	NP523776.1	POSH	9.000e-8	*Drosophila melanogaster*
	DN9152c0g2i1	XP001601412.2	scarface CG11066-PA	0.000009	*Nasonia vitripennis*
	DN20509c0g1i1	NP001123523.1	sortilin related receptor	2.000e-1	*Nasonia vitripennis*
	DN16049c0g1i1	NP001123523.1	sortilin related receptor	2.000e-7	*Nasonia vitripennis*
	DN11593c0g4i1	XP394101.2	SP16	0.000003	*Apis mellifera*

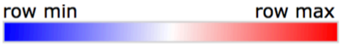

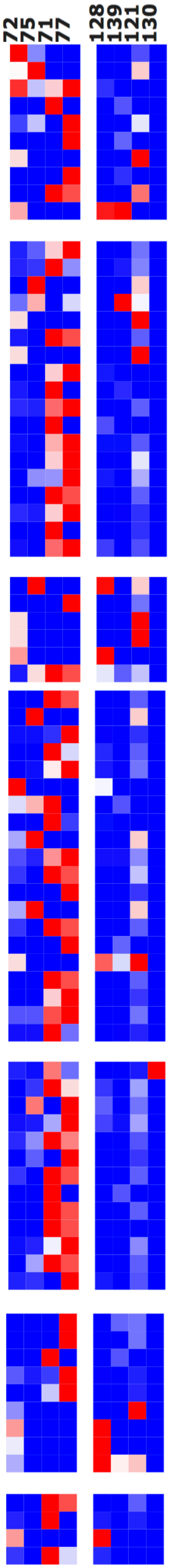

	Contig ID	Accession	Putative Function	E-value	Host
G	DN17216c0g1i1	ADR50356.1	prophenoloxidase	8.000e-26	*Oratosquilla oratoria*
	DN30261c0g1i1	CUS19247.1	prophenoloxidase	2.000e-3	*Argulus foliaceus*
	DN521c0g1i1	CUS19247.1	prophenoloxidase	1.000e-8	*Argulus foliaceus*
	DN23270c0g1i1	AFW98991.1	prophenoloxidase activating enzyme	3.000e-9	*Litopenaeus vannamei*
	DN6481c0g1i1	ABE03741.1	prophenoloxidase activating factor	9.000e-7	*Penaeus monodon*
	DN19381c0g1i1	AAX48010.1	prophenoloxidase, partial	4.000e-7	*Macrobrachium rosenbergii*
	DN22696c0g1i1	ACP19558.1	prophenoloxidase-activating enzyme	9.000e-8	*Penaeus monodon*
	DN45948c0g1i1	ACP19558.1	prophenoloxidase-activating enzyme	1.000e-37	*Penaeus monodon*
	DN1919c0g1i1	ACX47133.1	prophenoloxidase-activating factor	1.000e-16	*Portunus trituberculatus*
	DN37487c0g1i1	ACX47133.1	prophenoloxidase-activating factor	8.000e-1	*Portunus trituberculatus*
H	DN52506c0g1i1	AII16502.1	catalase, partial	3.000e-45	*Paracyclopina nana*
	DN33466c0g1i1	XP001606081.2	ENSANGP00000006233	3.000e-9	*Nasonia vitripennis*
	DN28036c0g1i1	XP001606463.1	ENSANGP00000012893	1.000e-26	*Nasonia vitripennis*
	DN12157c0g2i14	XP001600045.1	ENSANGP00000014839	2.000e-19	*Nasonia vitripennis*
	DN48812c0g1i1	AMV91696.1	glutathione peroxidase, partial	9.000e-15	*Portunus trituberculatus*
	DN20685c0g1i1	NP001166228.1	heat shock cognate 70 protein	6.000e-6	*Nasonia vitripennis*
	DN45216c0g1i1	AII16535.1	heat shock factor-binding protein 1, partial	2.000e-14	*Paracyclopina nana*
	DN13761c0g1i1	AHA61465.1	heat shock protein 70 cognate 3	1.000e-93	*Eriocheir sinensis*
	DN51752c0g1i1	AHA61465.1	heat shock protein 70 cognate 3	5.000e-47	*Eriocheir sinensis*
	DN812c0g1i1	NP001153522.1	heat shock protein cognate 4	1.000e-7	*Apis mellifera*
	DN50072c0g1i1	NP001153522.1	heat shock protein cognate 4	7.000e-27	*Apis mellifera*
	DN9138c0g2i1	NP001153544.1	heat shock protein Hsp70Ab-like	9.000e-115	*Apis mellifera*
	DN3719c0g2i1	XP001605191.2	Hsp90	7.000e-4	*Nasonia vitripennis*
	DN46194c0g1i1	XP001605191.2	Hsp90	4.000e-12	*Nasonia vitripennis*
	DN5391c0g1i1	XP001605191.2	Hsp90	6.000e-54	*Nasonia vitripennis*
	DN44359c0g1i1	AII16534.1	Hsp90, partial	3.000e-44	*Paracyclopina nana*
	DN745c0g1i1	XP001601130.1	mCG14932	5.000e-29	*Nasonia vitripennis*
	DN17443c0g1i1	AII16519.1	Mn SOD, partial	2.000e-1	*Paracyclopina nana*
I	DN53036c0g1i1	ACO15753.1	broad-complex core protein isoform 6	4.000e-13	*Caligus clemensi*
	DN15862c0g1i1	EFX85174.1	hindsight transcription factor-like protein	3.000e-8	*Daphnia pulex*
	DN28736c0g1i1	EFX85174.1	hindsight transcription factor-like protein	3.000e-8	*Daphnia pulex*
	DN42862c0g1i1	EFX85174.1	hindsight transcription factor-like protein	2.000e-7	*Daphnia pulex*
	DN20298c0g1i1	EFX85174.1	hindsight transcription factor-like protein	0.000007	*Daphnia pulex*
	DN12156c4g2i1	AGT03502.1	transcription factor btf3, partial	4.000e-3	*Procambarus clarkii*
J	DN21796c0g1i1	AAM73784.1	14-3-3 zeta-like type II, partial	6.000e-27	*Penaeus monodon*
	DN2598c0g2i1	AIE56158.1	calmodulin	1.000e-39	*Eriocheir sinensis*
	DN54232c0g1i1	AIE56158.1	calmodulin	3.000e-13	*Eriocheir sinensis*
	DN39127c0g1i1	AIE56158.1	calmodulin	4.000e-8	*Eriocheir sinensis*
	DN5807c0g1i1	JAM77015.1	carboxyl-terminal PDZ ligand; nitric oxide synthase	0.000006	*Daphnia magna*
	DN7083c0g1i2	JAM77015.1	carboxyl-terminal PDZ ligand; nitric oxide synthase	0.00001	*Daphnia magna*
	DN46632c0g2i1	AII16522.1	casein kinase I epsilon, partial	0.000004	*Paracyclopina nana*
	DN39744c0g1i1	AII16510.1	glutathione S-transferase delta-epsilon 2, partial	5.000e-8	*Paracyclopina nana*
	DN53924c0g1i1	AII16513.1	glutathione S-transferase mu3, partial	1.000e-35	*Paracyclopina nana*
	DN22070c0g1i1	ALP46200.1	nascent polypeptide-associated complex alpha	4.000e-27	*Eriocheir sinensis*
	DN52235c0g1i1	EFX77274.1	notch 2	3.000e-9	*Daphnia pulex*
	DN36402c0g1i1	EFX77274.1	notch 2	0.000004	*Daphnia pulex*
	DN13388c0g1i1	EFX77274.1	notch 2	3.000e-7	*Daphnia pulex*
	DN17061c0g1i1	AFI39876.1	phosphoglucose isomerase, partial	4.000e-26	*Daphnia pulex*
	DN28470c0g1i1	AII16498.1	protein phosphatase 1E, partial	6.000e-33	*Paracyclopina nana*
	DN10981c1g2i1	AIT40218.1	signal transducing adaptor molecule	2.000e-12	*Rhipicephalus microplus*
	DN53686c0g1i1	AAN77377.1	smooth endoplasmic reticulum calcium ATPase	2.000e-39	*Porcellio scaber*
	DN51815c0g1i1	AAN77377.1	smooth endoplasmic reticulum calcium ATPase	2.000e-11	*Porcellio scaber*
	DN180c0g1i1	AGC96525.1	thioredoxin 1	5.000e-37	*Scylla paramamosain*
	DN135c0g1i1	AIT40206.1	ubiquitin protein ligase Ubc5	4.000e-41	*Rhipicephalus microplus*
K	DN11630c1g2i1	ADG45306.1	actin	0	*Marsupenaeus japonicus*
	DN12114c1g3i8	ADG45308.1	actin	3.000e-4	*Marsupenaeus japonicus*
	DN2236c0g1i1	AII16466.1	cadherin, partial	2.000e-13	*Paracyclopina nana*
	DN10625c0g1i1	P35418.2	Paramyosin	4.000e-33	*Taenia sp.*
	DN20707c0g2i1	NP476950.1	Rac1	1.000e-56	*Drosophila melanogaster*
	DN24381c0g1i1	AMQ26208.1	Rac1	4.000e-58	*Litopenaeus vannamei*
	DN20796c0g1i1	AII16470.1	Ras-related protein Rab-2, partial	4.000e-8	*Paracyclopina nana*
	DN53867c0g1i1	ALJ96715.1	transglutaminase II	1.000e-12	*Eriocheir sinensis*
	DN170c0g2i1	Q25008.1	tubulin alpha-1 chain	1.000e-68	*Homarus americanus*
	DN26963c0g2i1	Q25008.1	tubulin alpha-1 chain	3.000e-26	*Homarus americanus*
	DN1793c0g1i1	Q94572.1	tubulin alpha-3 chain	6.000e-58	*Homarus americanus*
	DN30282c0g1i1	Q94572.1	tubulin alpha-3 chain	5.000e-31	*Homarus americanus*
	DN29549c0g1i1	Q94572.1	tubulin alpha-3 chain	3.000e-85	*Homarus americanus*
L	DN50124c0g1i1	AME17864.1	beta-arrestin1	3.000e-8	*Marsupenaeus japonicus*
	DN36972c0g1i1	AGL46986.1	C-type lectin	9.000e-2	*Procambarus clarkii*
	DN940c0g1i1	AGC96521.1	C-type lectin, partial	6.000e-31	*Scylla paramamosain*
	DN11857c0g2i3	AII16543.1	cubilin, partial	0.000005	*Paracyclopina nana*
	DN54008c0g1i1	AFI39732.1	enolase, partial	6.000e-56	*Daphnia parvula*
	DN44552c0g1i1	AGT03509.1	ferritin, partial	4.000e-1	*Procambarus clarkii*
	DN20843c0g1i1	CAB41634.1	iron regulatory protein 1-like protein	7.000e-9	*Pacifastacus leniusculus*
	DN25964c0g1i1	AII16462.1	PDZ and LIM domain protein 1, partial	4.000e-8	*Paracyclopina nana*
	DN2192c0g2i1	XP018012592.1	scavenger receptor class B member 1-like*	1.000e-12	*Hyalella azteca*
M	DN50607c0g1i1	XP018024334.1	cell cycle and apoptosis regulator protein 1-like*	7.000e-17	*Hyalella azteca*
	DN32791c0g1i1	XP018024334.1	cell cycle and apoptosis regulator protein 1-like*	2.000e-33	*Hyalella azteca*
	DN18405c0g1i1	JAN89210.1	cell division cycle protein	0.000002	*Daphnia magna*
	DN11227c1g2i1	XP018025155.1	cell division cycle protein 16 homolog*	9.000e-14	*Hyalella azteca*
