# Supplementary material for: Gene expression of benthic amphipods (genus: Diporeia) in relation to a circular ssDNA virus across two Laurentian Great Lakes
Source: PeerJ. 2017 Sep 26;5:e3810. doi: 10.7717/peerj.3810 (PMC5621510; doi:10.7717/peerj.3810)
Supplement: Supplemental Information 7 — Organisms were acquired via Ponar benthic sampler from the R/V Lake Guardian between August–September, 2014 (n = 98). RT-qPCR (n) refers to the number of samples per station allocated to RT-qPCR. HTS (high throughput sequencing) refers to stations where amphipods were collected for transcriptome preparation and sequencing. Haplotype was determined via cytochrome c oxidase I (COI) sequencing (Pilgrim et al., 2009). [file peerj-05-3810-s007.docx]

| **Lake** | **Station** | **Haplotype** | **Collection Date** | **Depth (m)** | **Latitude**  **(N)** | **Longitude**  **(W)** | **HTS** | **RT-qPCR (n)** |
| --- | --- | --- | --- | --- | --- | --- | --- | --- |
| Michigan | Mi27 | Southern | Aug. 7, 2014 | 102 | 43.60 | -86.916667 | ✓ | 21 |
| Michigan | Mi40 | Southern | Aug. 8, 2014 | 167.6 | 44.76 | -86.966667 | ✓ | 37 |
| Superior | Su23B | Northern | Aug. 24, 2014 | 63 | 46.8 | -84.80694 |  | 3 |
| Superior | Su067 | Northern | Aug. 25, 2014 | 81 | 46.6002 | -86.8204 |  | 2 |
| Superior | Su066 | Northern | Aug. 25, 2014 | 44 | 48.29727 | -88.96588 | ✓ | 12 |
| Superior | Su01 | Northern | Aug 24, 2014 | 95.4 | 46.99328 | 85.16093 |  | 9 |
| Huron | Hu48M | Southern | Aug. 10, 2014 | 112 | 45.27833 | -82.451667 |  | 7 |
| Huron | Hu38M | Southern | Aug. 11, 2014 | 135 | 44.74 | -82.06 |  | 2 |
