# Supplementary material for: Gene expression of benthic amphipods (genus: Diporeia) in relation to a circular ssDNA virus across two Laurentian Great Lakes
Source: PeerJ. 2017 Sep 26;5:e3810. doi: 10.7717/peerj.3810 (PMC5621510; doi:10.7717/peerj.3810)
Supplement: Supplemental Information 8 — Transcriptome assembly statistics. Read libraries were pooled and assembled de novo using de Bruijn graphs integrated into Trinity v.2.4.0, a tripartite assembly program (software modules: Inchworm, Chrysalis, and Butterfly) implemented on the Galaxy bioinformatics platform per default parameters (National Center for Genome Analysis Support, Indiana University Pervasive Technology Institute; Trinity –max_memory 240G –CPU 8 –normalize_reads –monitoring –seqType seq_type –single singlefile or –left left_file –right right_file). [file peerj-05-3810-s008.docx]

| **Total Number of contigs** | 82,074 |
| --- | --- |
| **Max contig length** | 10,795 |
| **Min contig length** | 201 |
| **Average contig length** | 309.78 |
| **Median contig length** | 261 |
| **Standard deviation of contig length** | 218.88 |
| **N50** | 290 |
