# Supplementary material for: Gene expression of benthic amphipods (genus: Diporeia) in relation to a circular ssDNA virus across two Laurentian Great Lakes
Source: PeerJ. 2017 Sep 26;5:e3810. doi: 10.7717/peerj.3810 (PMC5621510; doi:10.7717/peerj.3810)
Supplement: Supplemental Information 9 — Reference sequences fitting both keyword 1 and 2 (e.g. “Crustacea” + “Toll like receptor”) were collected and collated from the NCBI protein repository as a BLAST database to identify transcriptome contigs affiliated with putative immune functions. [file peerj-05-3810-s009.docx]

| **Keyword 1** | **Keyword 2** |
| --- | --- |
| *Crustacea* + | Immune |
|  | Toll like receptor |
|  | Toll |
|  | TLR |
|  | Prophenoloxidase |
|  | STAT |
|  | JNK |
|  | JAK |
|  | JAK/STAT |
|  | IMD |
|  | Humoral |
|  | Cell cycle |
|  | Antiviral |
|  | Antimicrobial peptides |
|  | Caspase |
|  | Chitinase |
|  | Nitric oxide synthase |
|  | DSCAM |
|  | Argonaute |
|  | Dicer |
|  | Dorsal |
|  | Gemini |
|  | IMD |
|  | MyD88 |
|  | STAT |
|  | Cactus |
|  | Pelle |
|  | Relish |
|  | Tube |
|  | c-type lectin |
|  | GNBP |
|  | Galectin |
|  | PGRP |
|  | TEP |
|  | Scavenger a |
