# Supplementary material for: Gene expression of benthic amphipods (genus: Diporeia) in relation to a circular ssDNA virus across two Laurentian Great Lakes
Source: PeerJ. 2017 Sep 26;5:e3810. doi: 10.7717/peerj.3810 (PMC5621510; doi:10.7717/peerj.3810)
Supplement: Supplemental Information 10 — All reactions were 25ul and included SsoAdvanced™ Universal Probes Supermix (Bio-Rad Laboratories, Hercules, CA, USA) with 2 μM primer/probe oligo (Eurofins Scientific, Luxembourg City, Luxembourg) per reaction. Reaction efficiencies of duplex reactions were comparable to those when reactions were run independently. Quantities of target amplicons were standardized by reference gene EF1A using the following equation: (TargetRT–TargetNRT)/(EF1ART–EF1ANRT), where RT and NRT indicate samples that have been reverse transcribed via Superscript III (Invitrogen, Carlsbad, CA, USA), or not reverse transcribed (no-RT control), respectively. LLOD specifies average lower limit of detection (Ct) across all runs containing the indicated primer/probe set and the corresponding amplicon copy number. Samples with Ct values > LLOD were designated “no detection” (negative). Average threshold Ct indicates ΔRn where quantity was determined (per StepOnePlus software v. 2.3; Foster City, CA, USA). [file peerj-05-3810-s010.docx]

| **Target** | **Sequence (5’ -> 3’)** | |
| --- | --- | --- |
| ACT  (β-actin) | Standard | ATACCGTGTTCAATGGGGTACTTCAGAGTAAGGATACCTCGCTTGCTCTGAGCCTCATCTCCGACGTAGGCGTCCTTCTGACCCATACCGA |
|  | Probe | [FAM]AAGGATACCTCGCTTGCTCTGA[BHQ1a] |
|  | F-primer | ACCGTGTTCAATGGGGTA |
|  | R-primer | GGTATGGGTCAGAAGGAC |
|  | LLOD ($\bar{x}$) | Ct of 38.62 corresponds to 56.2 amplicon copies |
|  | Threshold Ct ($\bar{x}$) | 0.0324 |
| UBQ  (Ubiquitin-conjugating enzyme E2) | Standard | ATGGCTCTCAAAAGAATTAACAAGGAACTCCAGGATCTTGGGCGTGACCCTCCCGCACAGTGCTCCGCGGGGCCTGTAGGCGATGACATGTTCCA |
|  | Probe | [FAM]GTGACCCTCCCGCACAGT[BHQ1a] |
|  | F-primer | GGCTCTCAAAAGAATTAACA |
|  | R-primer | GAACATGTCATCGCCTAC |
|  | LLOD ($\bar{x}$) | Ct of 37.35 corresponds to 48.2 amplicon copies |
|  | Threshold Ct ($\bar{x}$) | 0.0170 |
| NMHC  (Non-muscular myosin heavy chain) | Standard | AGACATTGTGAGCGGATTCCTGAAGCCTCGTATCAAGGTCGTCAGAGAGGCCGTCACCAAAGCGCAGAACAAGGAACAGGT |
|  | Probe | [FAM]AGGTCGTCAGAGAGGCCGTCACCA[BHQ1a] |
|  | F-primer | ACATTGTGAGCGGATTCCTG |
|  | R-primer | CTGTTCCTTGTTCTGCGCTT |
|  | LLOD ($\bar{x}$) | Ct of 35.35 corresponds to 29.8 amplicon copies |
|  | Threshold Ct ($\bar{x}$) | 0.0554 |
| EF1A  (Elongation factor-1α) | Standard | ACGATGACCTGAGCAAGGAAGCTCTCAGCCTCCTTGGCGGGATCGTTCTTGGAGTCGGAGGTGA |
|  | Probe | [HEX]CTCTCAGCCTCCTTGGCG[BHQ1a] |
|  | F-primer | GATGACCTGAGCAAGGAA |
|  | R-primer | ACCTCCGACTCCAAGAAC |
|  | LLOD ($\bar{x}$) | Ct of 40.63 corresponds to 41.4 amplicon copies |
|  | Threshold Ct ($\bar{x}$) | 0.0246 |
| Thermocycling parameters for targets ACT, UBQ, NMHC and EF1A include: 1 cycle at 95 °C x 5 min, followed by 60 cycles of 95 °C for 30s and 59 °C for 30s | | |
| *LM29173-REP | Standard | TATTCTCCCGTTCGTTACTCCAGGTCTTTCTGATGCTGAACGAATCGGTGACACAATTAATCTTCGTTCTATGAGTTTGATGGGTCATA |
|  | Probe | [FAM]TGCTGAACGAATCGGTGACACAA[TAMRA] |
|  | F-primer | TTCTCCCGTTCGTTACTCCA |
|  | R-primer | TGACCCATCAAACTCATAGAAC |
|  | LLOD ($\bar{x}$) | Ct of 41.19 corresponds to 42.29 amplicon copies |
|  | Threshold Ct ($\bar{x}$) | 0.0382 |
| Thermocycling parameters for LM29173-REP include: 1 cycle at 95 °C x 5 min, followed by 60 cycles of 95 °C for 30s and 58 °C for 30s | | |
