# Supplementary material for: Gene expression of benthic amphipods (genus: Diporeia) in relation to a circular ssDNA virus across two Laurentian Great Lakes
Source: PeerJ. 2017 Sep 26;5:e3810. doi: 10.7717/peerj.3810 (PMC5621510; doi:10.7717/peerj.3810)
Supplement: Supplemental Information 11 — Reads were trimmed using CLC workbench (v. 8.5.1, Qiagen, Hilden, Germany: quality limit 0.05, no ambiguous nucleotides, maximum read length 251 nt, discard reads <50 nt), and assembled de novo using Trinity on the Galaxy bioinformatics platform per default parameters (National Center for Genome Analysis Support, Indiana University Pervasive Technology Institute, USA). [file peerj-05-3810-s011.docx]

| **Library** | **Viral Load Class** | **LM29173 Load/ Animal** | **Weight, mg** | **Total Reads** | **Reads after trimming** | **Ave Length after trimming** | **rRNA reads** | **Mapped Reads (total %)** |
| --- | --- | --- | --- | --- | --- | --- | --- | --- |
| SU066(363) | Low | 0.00 | 2.1 | 1,695,330 | 1,695,258 | 116.8 | 496,001 | 49.92 |
| SU066(361) | Low | 0.00 | 3.0 | 2,530,390 | 2,529,456 | 100.98 | 540,439 | 52.91 |
| SU066(359) | High | 6657.1 | 3.5 | 2,362,078 | 2,360,580 | 132.99 | 388.304 | 57.77 |
| SU066(358) | High | 12147.8 | 2.8 | 1,369,426 | 1,369,320 | 112.56 | 349,744 | 48.21 |
| MI27(72) | Low | 103808.3 | 0.2 | 1965884 | 1965175 | 165.795 | 378826 | 58.44 |
| MI27(75) | Low | 305707.0 | 0.2 | 593,998 | 593,680 | 81.3 | 88,653 | 62.59 |
| MI27(71) | High | 784921.8 | 3.4 | 800,136 | 800,122 | 81.14 | 295,642 | 56 |
| MI27(77) | High | 1735415.6 | 2.7 | 755,194 | 755,178 | 84.5 | 261,510 | 52.79 |
| MI40(128) | Low | 103116.0 | 1.0 | 975,640 | 975,104 | 80.53 | 138,837 | 58.13 |
| MI40(139) | Low | 139830.4 | 2.1 | 750,394 | 750,142 | 87.66 | 15,726 | 61.33 |
| MI40(121) | High | 3138098.4 | 0.8 | 886,256 | 886,112 | 80.39 | 144,175 | 58.53 |
| MI40(130) | High | 3197003.1 | 1.1 | 22,732 | 22,732 | 87.81 | 492 | 16.27 |
| Totals |  |  | 1.9  (ave) | 14,707,458 | 14,702,859 | 101.038  (ave) | 2,710,433 | 52.74%  (ave) |
